# Contrast-enhanced ultrasound findings of adult renal cell carcinoma associated with Xp11.2 translocation/TFE3 gene fusion: comparison with clear cell renal cell carcinoma and papillary renal cell carcinoma

**DOI:** 10.1186/s40644-019-0268-7

**Published:** 2019-12-31

**Authors:** Shuping Wei, Fuli Tian, Qiuyuan Xia, Pengfei Huang, Yidan Zhang, Zhichao Xia, Min Wu, Bin Yang

**Affiliations:** 1 Department of Ultrasound, Nanjing Drum Tower Hospital, The Affiliated Hospital of Nanjing University Medical School, 321 Zhongshan Road, Nanjing, 210008 Jiangsu China; 2 Department of Ultrasound, Jinling Hospital, Medical School of Nanjing University, 305 Zhongshan East Road, Nanjing, 210002 Jiangsu China; 3 Department of Pathology, Jinling Hospital, Medical School of Nanjing University, Nanjing, Jiangsu China; 4 Department of Domestic clinical application, Mindray Bio-Medical Electronics Co. Ltd, Shenzhen, Guangdong China

**Keywords:** Xp11.2 translocation, Contrast-enhanced ultrasound, Clear cell renal cell carcinoma, Papillary renal cell carcinoma, Differential diagnosis

## Abstract

**Background:**

To investigate the contrast-enhanced ultrasound (CEUS) findings of renal cell carcinoma (RCC) associated with Xp11.2 translocation/TFE3 gene fusion (Xp11.2/TFE3) in adult patients by comparison with those of clear cell RCC (ccRCC) and papillary RCC (pRCC).

**Methods:**

In total, 110 patients (110 renal masses) who underwent CEUS examinations were enrolled in this study. The cases included 18 Xp11.2/TFE3 RCCs, 60 ccRCCs and 32 pRCCs. All masses were confirmed by operative pathology. The CEUS imaging data of these patients were retrospectively analysed by two readers. The conventional US and CEUS features of Xp11.2/TFE3 RCC were compared with those of ccRCC and pRCC.

**Results:**

The age of the patients with Xp11.2/TFE3 RCC ranged from 20 to 68 years, with a mean age of 38.3 ± 16.3 years and a slight female predominance. The weighted kappa value that interprets the concordance between the interobserver agreement of the US and CEUS features ranged from 0.61 to 0.89. On conventional US and CEUS imaging of Xp11.2/TFE3 RCCs, the tumours were hypoechoic (6/18, 33.3%), isoechoic (8/18, 44.4%), and hyperechoic (4/18, 22.2%). The cystic component was present in 5 cases (27.8%), calcification was present in 9 cases (50.0%), and colour flow signal was present in 7 cases (38.9%). Most cases showed simultaneous wash-in (11/18, 61.1%); the peak enhancement showed hypoenhancement (6/18, 33.3%), isoenhancement (10/18, 55.6%), and hyperenhancement (2/18, 11.1%); most cases exhibited heterogeneous enhancement (12/18, 66.7%) and fast- or simultaneous-out (16/18, 88.9%); and a pseudocapsule was present in 6 cases (33.3%). In the multivariate logistic regression analysis, calcification and lower peak enhancement were more likely to be present in Xp11.2/TFE3 RCC than in ccRCC (*P* < 0.05), and younger age and relatively high peak enhancement were more likely to be present in Xp11.2/TFE3 RCC than in pRCC (*P* < 0.05). The calcification combined peak enhancement model differentiated Xp11.2/TFE3 RCC from ccRCC, and the age combined peak enhancement model differentiated Xp11.2/TFE3 RCC from pRCC with an AUC, a sensitivity and a specificity of 0.896, 94.4% and 73.3% and 0.786, 50.0% and 100.0%, respectively.

**Conclusions:**

The specific CEUS features combined with demographic information and clinical symptoms may be helpful for differentiating Xp11.2/TFE3 RCC from ccRCC and pRCC.

## Background

Renal cell carcinoma (RCC) associated with Xp11.2 translocation/TFE3 gene fusion (Xp11.2/TFE3) is an uncommon subtype of RCC. It was first considered as a genetically distinct entity of renal tumours according to the World Health Organization (WHO) classification in 2004 [1]. This renal cell tumour is primarily found in children and adolescents, accounting for 20%~ 40% of paediatric RCCs, but in adults it is very rare, the incidence was reported to be approximately 1–1.6% of all renal tumours [2]. However, it seems that these tumours in adults are generally more advanced and aggressive than in children. Additionally, tumours are frequently associated with local lymph node metastasis and distant metastasis, and the prognosis of Xp11.2/TFE3 RCC is poorer than that of the other subtypes of RCC [3].

Many radiologic reports have recently focused on the imaging findings of Xp11.2/TFE3 RCC [4, 5]. In recent years, contrast-enhanced ultrasound (CEUS) has been widely used to diagnose renal masses [6]. Compared with contrast-enhanced computed tomography (CT) or magnetic resonance imaging (MRI), there are many unique advantages of CEUS, such as cost-effectiveness, non-invasive, real-time imaging ability, no ionizing radiation, and the use of microbubble-based contrast agents with no nephrotoxicity, which make CEUS very valuable in the diagnosis of renal lesions. However, due to the low incidence of Xp11.2/TFE3 RCC, there are only a few previous reports that focusing on its CEUS characteristics to date, even those including children and adult patients together [7]. In consideration of the more advanced clinical stages and unfavourable courses in adult patients, in the present study, we focused on analysing the CEUS features of Xp11.2/TFE3 RCC in adult patients. Since clear cell RCC (ccRCC) is the most common renal carcinoma in adults, it should be differentiated from Xp11.2/TFE3 RCC first; moreover, papillary RCC (pRCC), which is the second most common subtype of RCC, often cannot be distinguished from Xp11.2/TFE3 RCC in routine histopathological examination. These two subtypes of RCC should be primarily considered for differentiation from Xp11.2/TFE3 RCC. Thus, in our study, the CEUS findings of Xp11.2/TFE3 RCC were further compared with those of ccRCC and pRCC to investigate the specific CEUS features for the diagnosis of Xp11.2/TFE3 RCC.

## Materials and methods

### Patients

This study was approved by the Institutional Review Board of Jinling Hospital, Medical School of Nanjing University. The requirement for informed consent was waived for this retrospective analysis of an observational registry. From May 2008 to June 2018, a total of 18 adult patients (8 males and 10 females; age range, 20–68 years; mean age, 38.3 ± 16.3 years) with proven Xp11.2/TFE3 RCC who underwent CEUS examinations preoperatively at Jinling Hospital, Medical School of Nanjing University (Nanjing, Jiangsu, China), were retrospectively included in our study. All the tumours were solitary and were diagnosed with pathology based on the morphological findings as well as immunohistochemical and molecular genetic findings (fluorescence in situ hybridization, FISH). To establish ccRCC and pRCC cohorts, we searched for adult patients with ccRCC and pRCC who also underwent CEUS examination preoperatively at the same hospital during that same time period (from May 2008 to June 2018). Patients with multiple renal masses were excluded from participation. Therefore, a total of 60 patients with ccRCC (age range, 23–78 years; mean age, 55.8 ± 12.8 years) and 32 patients with pRCC (age range, 30–75 years; mean age, 57.7 ± 11.0 years) were enrolled in this study. Overall, this study included 110 patients with 110 renal masses, and all patients were confirmed by histopathological diagnosis after surgical operation. The medical records and diagnostic imaging data of these patients were retrospectively analysed.

### Conventional US and CEUS imaging

All conventional US and CEUS examinations were performed using one of three US systems: Sequoia 512 (Siemens, Mountain View, CA, USA), equipped with a contrast pulse sequence (CPS) imaging mode (3–4 MHz convex probe, a low mechanical index in the range from 0.18 to 0.20); Resona 7 or DC-80 (Mindray, Shenzhen, China), both equipped with an ultra-wideband nonlinearity (UWN^+^) imaging mode (1–5 MHz convex probe, a low mechanical index of 0.07 or 0.10, respectively). The methods were similar to those described in our previous study [8]. In brief, the initial greyscale imaging mode was first used to observe the location, size, and echogenicity of the lesions, and the blood flow of the lesions was evaluated using colour Doppler flow imaging (CDFI), then contrast imaging mode was used, and a 1.6–2.4 ml dose of contrast agent (SonoVue; Bracco, Milan, Italy) was administered via intravenous injection in a bolus fashion and followed by a 5 ml of normal saline flush. When the CEUS examination was conducted, all patients were instructed to keep breathing slow and shallow, and the complete scanning was continued for at least 3 min. In this study, the contrast phases were classified into the phase terms as previous studies described: cortical phase, beginning 10–15 s after contrast agent injection until 30–45 s; medullary phase, beginning 30–45 s after contrast agent injection until the echoes of microbubbles completely disappeared [9–11]. Finally, all the CEUS images and cine loops were digitally stored for further analysis. All US and CEUS scanning procedures were performed by two investigators who both had more than 5 years of experience in abdominal CEUS studies.

### Imaging interpretation

The conventional US and CEUS images were retrospectively reviewed in a random order by two independent ultrasonologists who both had more than 10 years of experience in renal US and CEUS evaluation, the two readers both blinded to the clinical histories, any other cross-sectional imaging findings such as CT/MR, and the final histological results. The cases that the readers disagreed were re-evaluated by both and a final consensus result was obtained. The conventional US imaging characteristics, including the echogenicity, cystic component, presence of calcification and colour flow signals, were evaluated. The echogenicity of lesions was divided into hypoechoic, isoechoic, and hyperechoic regions compared with the renal cortex. The CEUS imaging characteristics were also evaluated, including the wash-in and wash-out patterns, the peak enhancement degree, the homogeneity of enhancement, and the pseudocapsule. The wash-in pattern was classified into slow-in and simultaneous-in, and the wash-out pattern was classified into fast- or simultaneous-out and slow-out. The degree of tumour at peak enhancement was divided into hypo-, iso-, and hyperenhancement compared with the adjacent renal cortex [12]. The homogeneity of enhancement was classified into homogeneous and heterogeneous [13]. The pseudocapsule was defined as a perilesional rim enhancement; in the early corticomedullary phase, it presented as a hypoechoic rim; in the late nephrographic phase, it would become hyperechoic and more distinct [14].

### Statistical analysis

SPSS 16.0 (SPSS, Inc., Chicago, IL) and MedCalc Software (MedCalc, Mariakerke, Belgium) were used for statistical analysis. The data was expressed as the mean (standard deviation). To analyse the differences between groups, Student’s t-test was used for continuous variables, and the chi-square test and Fisher’s exact test were used for categorical variables. To interpret the concordance between interobserver agreement, the weighted Kappa was used, the Kappa values were interpreted as follows: 0–0.20, poor agreement; 0.21–0.40, fair agreement; 0.41–0.60, moderate agreement; 0.61–0.80, good agreement; and 0.81–1.00, excellent agreement. The imaging parameters that emerged as significant differentiators in univariate analysis were used in multivariate logistic regression analysis for differentiating Xp11.2/TFE3 RCC from ccRCC and pRCC. To evaluate the diagnostic performance of the logistic regression models in the differentiation of Xp11.2/TFE3 RCC from ccRCC and pRCC, receiver operating characteristic (ROC) curves were used, the area under the curve (AUC) and corresponding sensitivity and specificity with 95% confidence intervals (CIs) were calculated. A *P*-value that less than 0.05 was considered to be statistically significant.

## Results

### Patient and tumour characteristics

The clinical characteristics of the 18 patients with Xp11.2/TFE3 RCC are summarized in an additional file to show these characteristics in more detail (see Additional file 1). In total, 7 of 18 patients (38.9%) presented with symptoms, including gross haematuria, flank pain and fever, whereas 11 cases (61.1%) were detected incidentally. Follow-up information was available for 15 cases (83.3%), and after a mean follow-up interval of 17.9 months (range 5–52 months), 3 patients were found with recurrence or metastases, and 12 were alive without recurrence or metastases.

Table 1 describes the patients and tumour characteristics of Xp11.2/TFE3 RCC, ccRCC and pRCC. Xp11.2/TFE3 RCC more frequently affected young women; of these 18 patients, 11 patients (61.1%) were younger than 40 years old, and 7 patients (38.9%) were older than 40 years old, while ccRCC and pRCC more frequently affected older men. In the univariate analysis, there were significant differences in age and gender between Xp11.2/TFE3 RCC and ccRCC (*P* < 0.05), and there was a significant difference in age between Xp11.2/TFE3 RCC and pRCC (*P* < 0.05). However, no significant difference was found in the size, tumour side or tumour location between Xp11.2/TFE3 RCC and ccRCC or between Xp11.2/TFE3 RCC and pRCC (*P* > 0.05).

**Table 1:** Patient and tumor characteristics in the three subtypes of RCC

	Xp11.2/TFE3 RCC (*n* = 18)	ccRCC (*n* = 60)	pRCC (*n* = 32)	*P* value	
				Xp11.2/TFE3 RCC vs ccRCC	Xp11.2/TFE3 RCC vs pRCC
Age (years)	38.3 ± 16.3	55.8 ± 12.8	57.7 ± 11.0	0.049*	0.007*
Gender no. (%)				0.033*	0.055
Male	8 (44.4)	43 (71.7)	23 (71.9)		
Female	10 (55.6)	17 (28.3)	9 (28.1)		
Size (cm)	4.3 ± 1.5	3.3 ± 1.5	3.6 ± 1.7	0.904	0.409
Tumor side (%)				0.561	0.585
Left	7 (38.9)	28 (46.7)	15 (46.9)		
Right	11 (61.1)	32 (53.3)	17 (53.1)		
Tumor location (%)				0.217	0.512
Upper pole	8 (44.4)	16 (26.7)	12 (37.5)		
Interpolar pole	5 (27.8)	14 (23.3)	6 (18.8)		
Lower pole	5 (27.8)	30 (50.0)	14 (43.7)		

* Significant value

### Interobserver agreement

The US and CEUS features of these three RCC subtypes evaluated by Reader 1 and Reader 2 are summarized in an additional file to show these findings in more detail (see Additional file 2). There was good to excellent agreement between the readers for the US and CEUS features, as evidenced by kappa statistics that ranged from 0.61 to 0.89.

### Conventional US and CEUS features

**Fig. 1:**
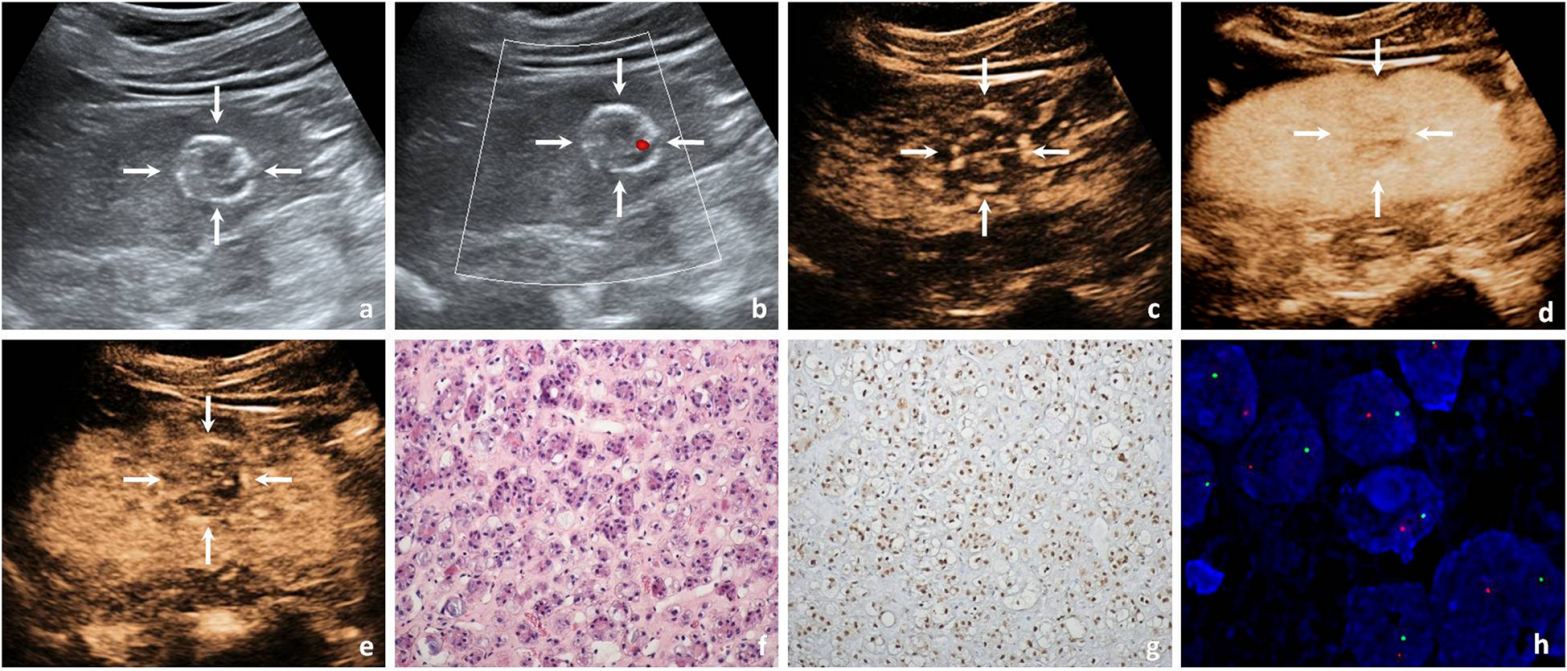
A 68-year-old man with a 2.5-cm diameter Xp11.2/TFE3 RCC. **a** Conventional US showed a hypoechoic mass with peripheral calcifications in the interpolar region of the right kidney (arrows). **b** CDFI showed the tumour lack of blood flow signal (arrows). **c** The tumour enhanced simultaneously with the renal cortex at the initial enhancement of CEUS imaging (arrows). **d** The tumour showed heterogeneous hypoenhancement at the peak enhancement of CEUS imaging (arrows). **e** The tumour showed fast wash-out at the medullary phase of CEUS imaging (arrows). **f** Haematoxylin eosin (HE) staining showed an eosinophilic cytoplasm arranged with a nested architecture (× 200). **g** Immunohistochemical staining demonstrated diffuse and strong TFE3 protein expression in the tumour cell nucleus (× 200). **h** FISH showed separated red and green signals in the tumour cell nucleus, which were considered to be the positive signals of TFE3 rearrangement (× 1000)

On conventional US imaging of Xp11.2/TFE3 RCC, 6 cases (33.3%) were hypoechoic, 8 cases (44.4%) were isoechoic, and 4 cases (22.2%) were hyperechoic. The cystic component was present in 5 cases (27.8%), calcification was present in 9 cases (50.0%), and colour flow signal was present in 7 cases (38.9%). Thrombosis was found in the right renal vein and inferior vena cava in only one case (5.6%, 1/18). On CEUS imaging of Xp11.2/TFE3 RCC, most cases showed simultaneous wash-in (61.1%). At peak enhancement, 6 cases (33.3%) showed hypoenhancement, 10 cases (55.6%) showed isoenhancement, and 2 cases (11.1%) showed hyperenhancement; most cases showed heterogeneous enhancement (66.7%) and fast- or simultaneous-out (88.9%), while the pseudocapsule was present in only 6 cases (33.3%) (Fig. 1, Table 2).

**Table 2:** The differences in conventional US and CEUS features among the three subtypes of RCC

Features	Xp11.2/TFE3 RCC (*n* = 18)	ccRCC (*n* = 60)	pRCC (*n* = 32)	*P* value	
				Xp11.2/TFE3 RCC vs ccRCC	Xp11.2/TFE3 RCC vs pRCC
Echogenicity (%)				0.605	0.176
Hypoechoic	6 (33.3)	28 (46.7)	17 (53.1)		
Isoechoic	8 (44.4)	21 (35.0)	13 (40.6)		
Hyperechoic	4 (22.2)	11 (18.3)	2 (6.3)		
Cystic component(%)				0.413	0.830
Present	5 (27.8)	23 (38.3)	8 (25.0)		
Absent	13 (72.2)	37 (61.7)	24 (75.0)		
Calcification (%)				< 0.001*	0.009*
Present	9 (50.0)	4 (6.7)	5 (15.6)		
Absent	9 (50.0)	56 (93.3)	27 (84.4)		
Color flow signal (%)				0.087	0.198
Present	7 (38.9)	37 (61.7)	7 (21.9)		
Absent	11 (61.1)	23 (38.3)	25 (78.1)		
Wash-in(%)				0.001*	0.022*
Slow-in	7 (38.9)	3 (5.0)	23 (71.9)		
Simultaneous-in	11 (61.1)	57 (95.0)	9 (28.1)		
Peak Enhancement (%)				< 0.001*	0.004*
Hypoenhancement	6 (33.3)	3 (5.0)	24 (75.0)		
Isoenhancement	10 (55.6)	10 (16.7)	4 (12.5)		
Hyperenhancement	2 (11.1)	47 (78.3)	4 (12.5)		
Homogeneity (%)				0.683	0.077
Homogeneous	6 (33.3)	17 (28.3)	19 (59.4)		
Heterogeneous	12 (66.7)	43 (71.7)	13 (40.6)		
Wash-out (%)				0.354	0.602
Fast- or simultaneous-out	16 (88.9)	45 (75.0)	31 (96.9)		
Slow-out	2 (11.1)	15 (25.0)	1 (3.1)		
Pseudocapsule (%)				0.449	0.254
Present	6 (33.3)	26 (43.3)	16 (50.0)		
Absent	12 (66.7)	34 (56.7)	16 (50.0)		

* Significant value

**Fig. 2:**
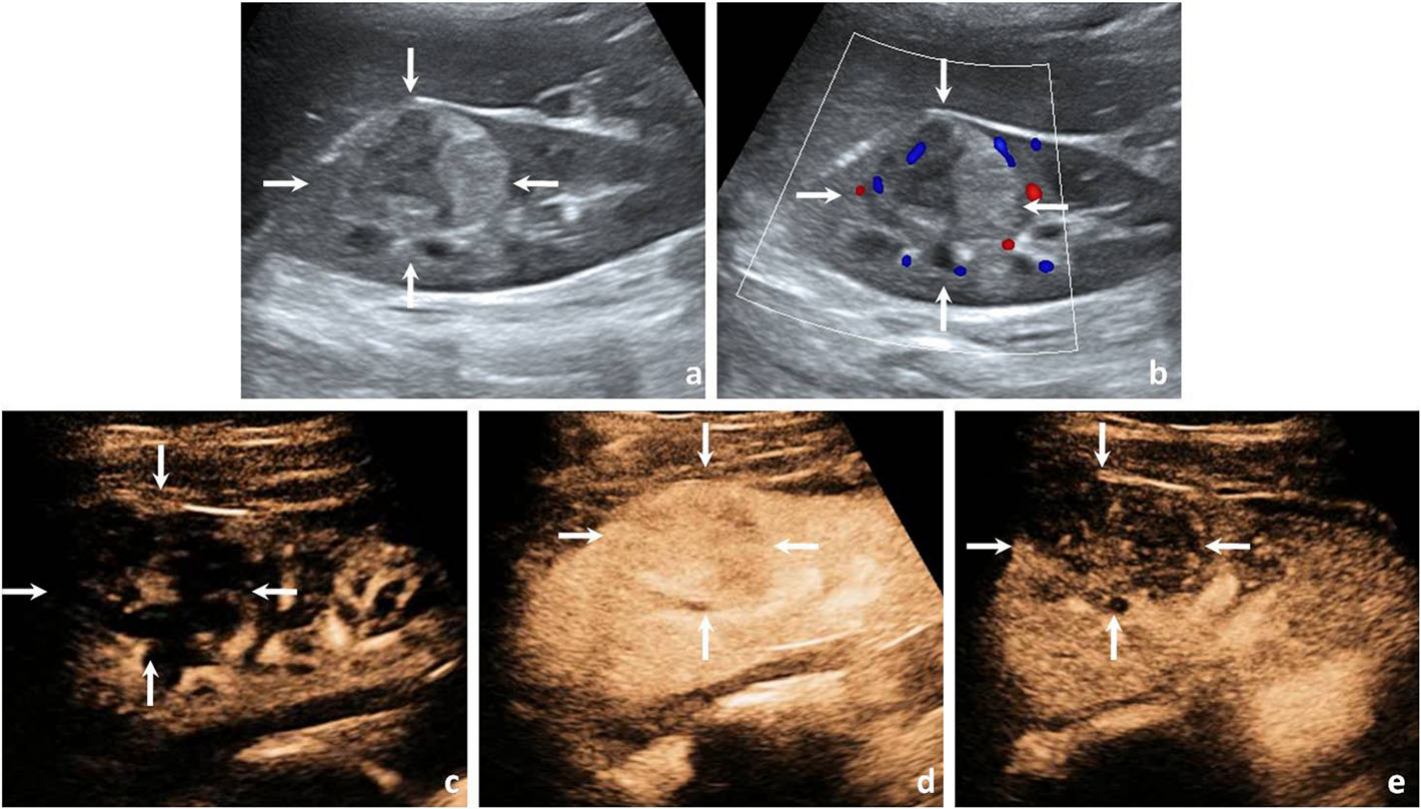
A 24-year-old woman with a 4.2-cm diameter Xp11.2/TFE3 RCC. **a** Conventional US showed an isoechoic mass in the upper pole of the right kidney (arrows). **b** CDFI revealed a few blood flow signals around the tumour periphery (arrows). **c** The tumour enhanced simultaneously with the renal cortex at the initial enhancement of CEUS imaging (arrows). **d** The tumour showed heterogeneous isoenhancement at the peak enhancement of CEUS imaging (arrows). **e** The tumour showed fast wash-out at the medullary phase of CEUS imaging (arrows)

**Fig. 3:**
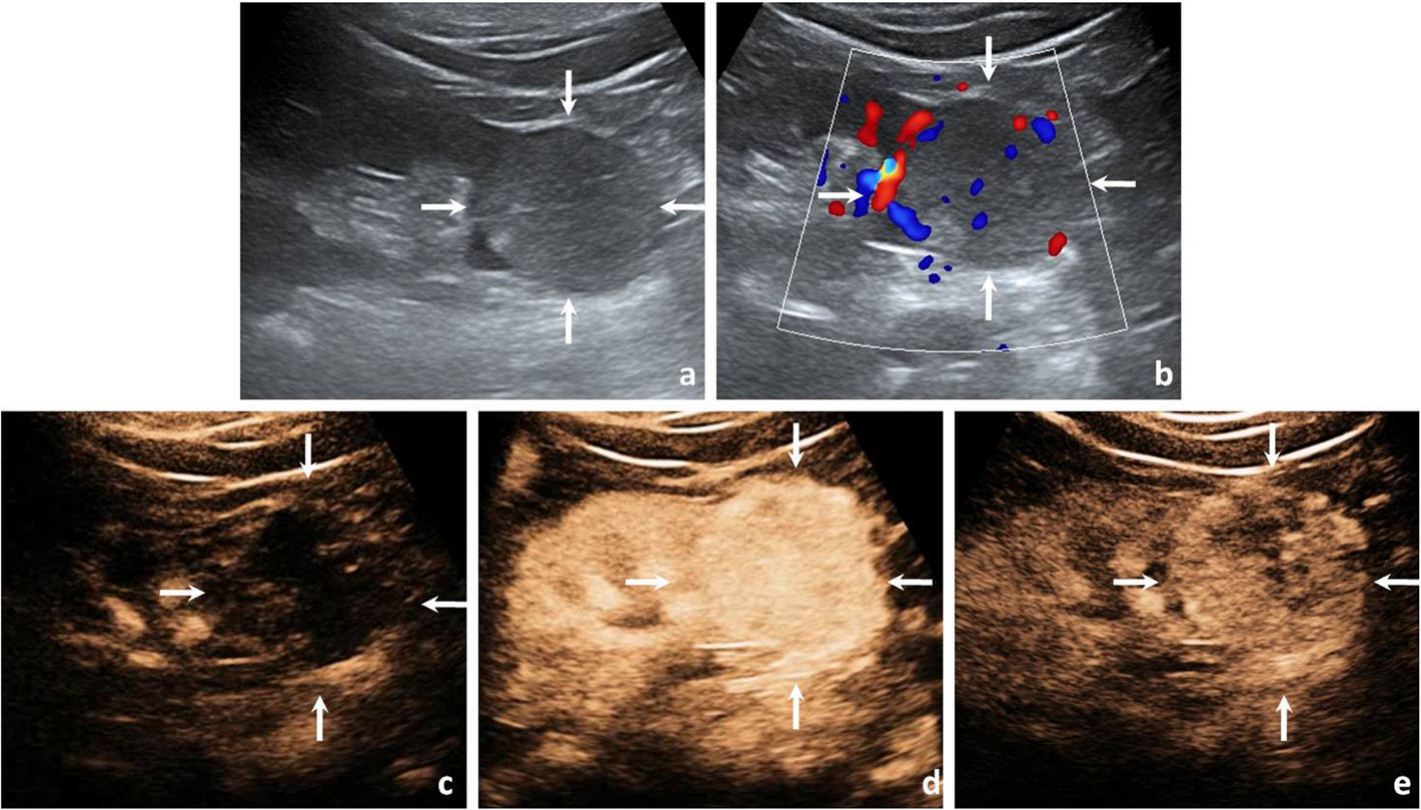
A 58-year-old woman with a 4.9-cm diameter ccRCC. **a** Conventional US showed an isoechoic mass in the lower pole of the left kidney (arrows). **b** CDFI revealed some blood flow signals around and within the tumour (arrows). **c** The tumour enhanced simultaneously with the renal cortex at the initial enhancement of CEUS imaging (arrows). **d** The tumour showed heterogeneous prominent hyperenhancement, with perilesional rim enhancement at the peak enhancement of CEUS imaging (arrows). **e** The wash-out feature of the tumour was similar to that of the renal cortex at the medullary phase of CEUS imaging (arrows)

**Fig. 4:**
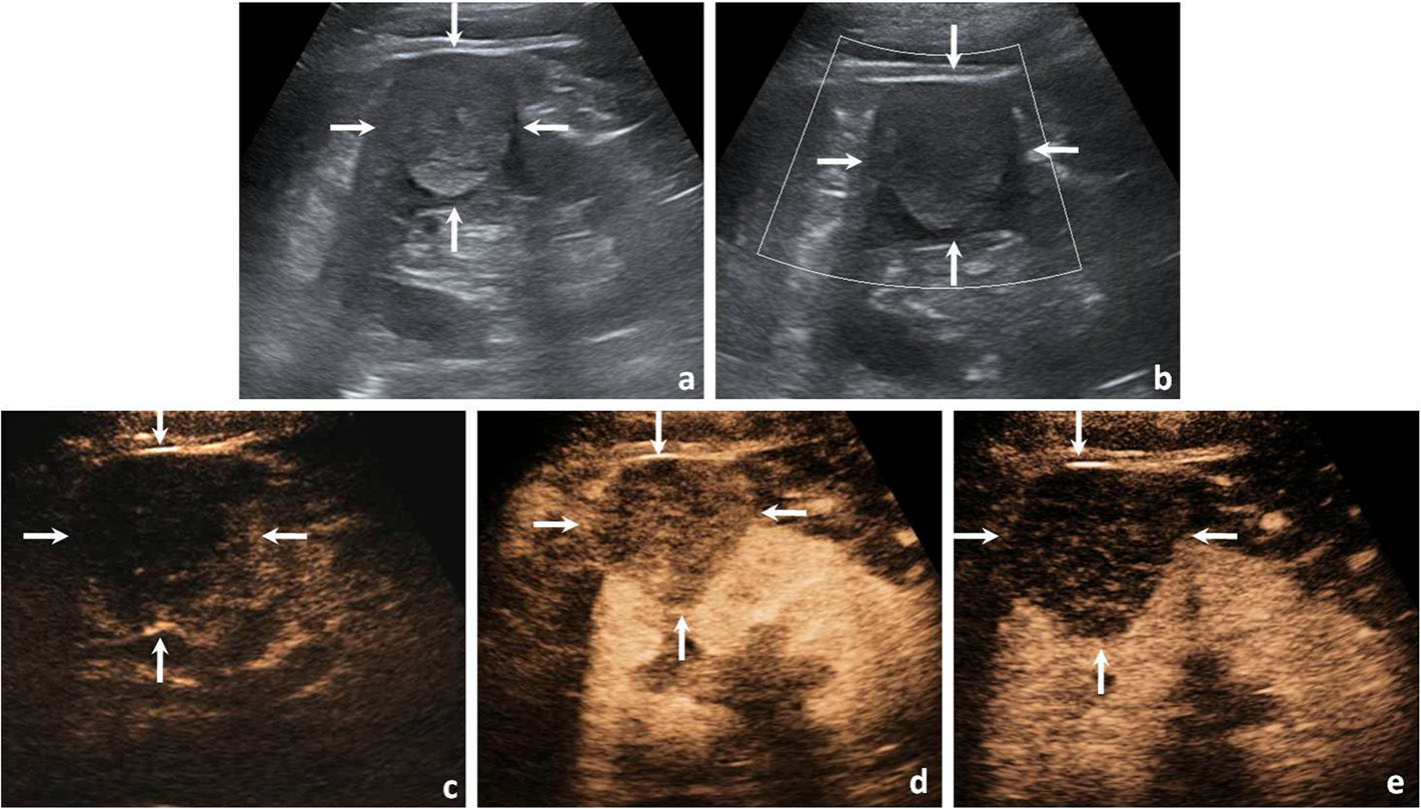
A 46-year-old man with a 4.5-cm diameter pRCC. **a** Conventional US showed an isoechoic mass in the upper polar pole of the left kidney (arrows). **b** CDFI showed the tumour lack of blood flow signals (arrows). **c** The tumour enhanced slower than the renal cortex at the initial enhancement of CEUS imaging (arrows). **d** The tumour showed homogenous hypoenhancement at the peak enhancement of CEUS imaging (arrows). **e** The tumour showed fast wash-out at the medullary phase of CEUS imaging (arrows)

In the univariate analysis comparing Xp11.2/TFE3 RCC with ccRCC and pRCC, calcification was more common in Xp11.2/TFE3 RCC than in ccRCC and pRCC (both *P* < 0.05). As simultaneous wash-in was observed in the majority of ccRCCs (95.0%), in the wash-in pattern, there was a significant difference between Xp11.2/TFE3 RCC and ccRCC (*P* < 0.05) and between Xp11.2/TFE3 RCC and pRCC (*P* < 0.05). For peak enhancement, most ccRCCs (78.3%) showed hyperenhancement, and most pRCCs (75.0%) showed hypoenhancement. There was a significant difference in peak enhancement between Xp11.2/TFE3 RCC and ccRCC (*P* < 0.05) and between Xp11.2/TFE3 RCC and pRCC (*P* < 0.05) (Figs. 2, 3, 4).

### Multivariate analysis and ROC analysis

In a multivariate logistic regression analysis with age, calcification and peak enhancement in the model, we found two significant predictors for differentiating Xp11.2/TFE3 RCC from ccRCC: the presence of calcification and lower peak enhancement were more likely in Xp11.2/TFE3 RCC (*P* < 0.05). Moreover, there were also two significant predictors for differentiating Xp11.2/TFE3 RCC from pRCC: younger age (less than 50.7 years) and relatively higher peak enhancement were more likely to be present in Xp11.2/TFE3 RCC (*P* < 0.05). Table 3 lists the significant predictors and their odds ratios (ORs).

**Table 3:** Multivariate logistic regression analyses of ultrasound parameters differentiating Xp11.2/TFE3 RCC from ccRCC and pRCC

Variable	Xp11.2/TFE3 RCC vs ccRCC		Xp11.2/TFE3 RCC vs pRCC	
	*P* value	OR (95% CI)	*P* value	OR (95% CI)
Age^a^	–	–	0.010	6.894 (1.582–30.046)
Calcification	0.006	12.915 (2.066–80.721)	–	–
Peak enhancement	< 0.001	27.485 (4.718–160.116)	0.006	7.961 (1.826–34.713)

Dash indicates not significant

^a^Age were classified on a scale of grade 1–2: grade 1, age was younger than the mean age of the whole two groups; grade 2, age was elder than the mean age of the whole two groups. The mean age of Xp11.2/TFE3 RCC and ccRCC was 51.7 years, the mean age of Xp11.2/TFE3 RCC and pRCC was 50.7 years

**Fig. 5:**
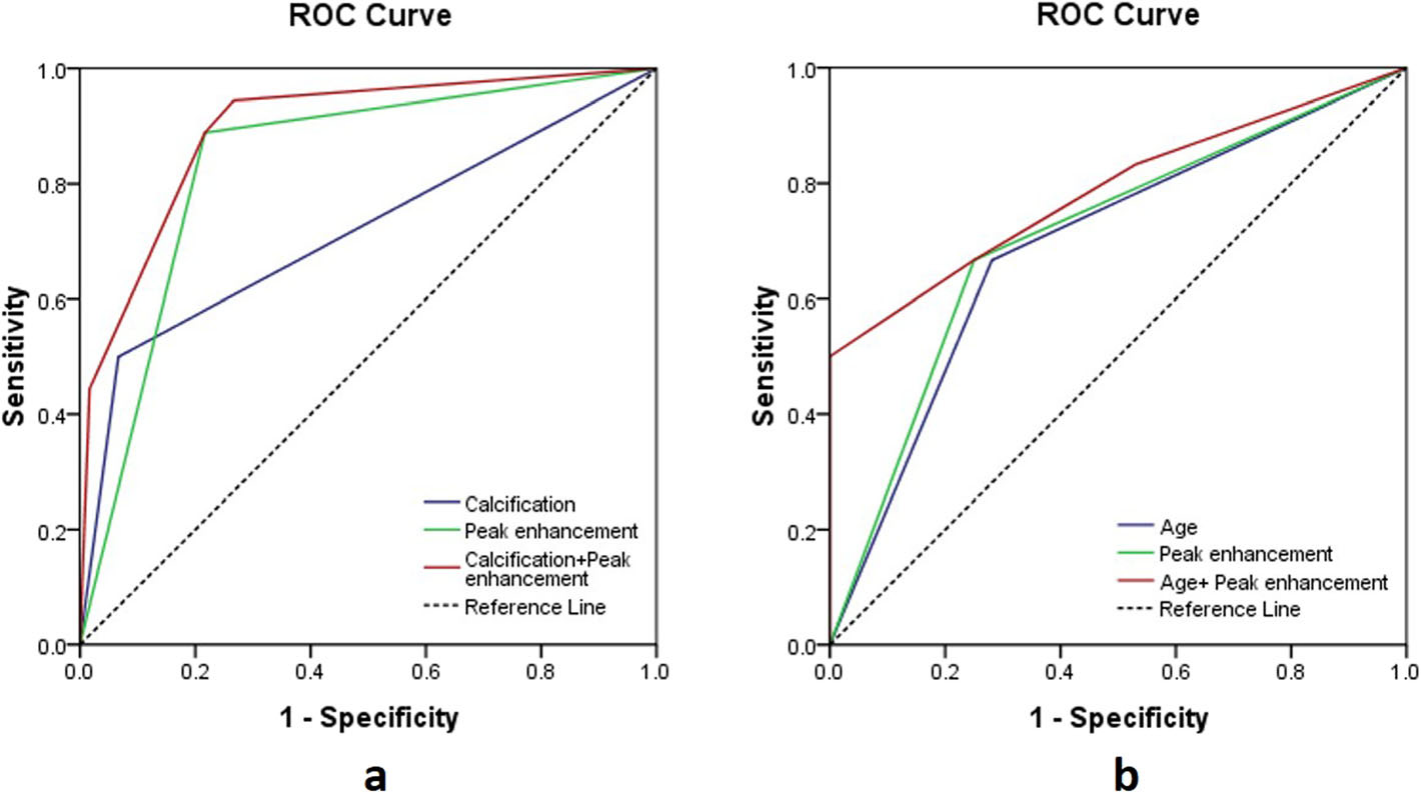
ROC curves of the logistic regression models for the differentiation of Xp11.2/TFE3 RCC from ccRCC (**a**) and pRCC (**b**). The AUCs are shown in Table 4

The AUCs, sensitivities and specificities with 95% CIs were extracted from ROC curve analysis (Fig. 5) and are summarized in Table 4. The calcification combined peak enhancement model differentiated Xp11.2/TFE3 RCC from ccRCC, and the age combined peak enhancement model differentiated Xp11.2/TFE3 RCC from pRCC with an AUC, a sensitivity and a specificity of 0.896, 94.4% and 73.3% and 0.786, 50.0% and 100.0%, respectively.

**Table 4:** Diagnostic performance of logistic regression models in differentiation of Xp11.2/TFE3 RCC from ccRCC and pRCC

	AUC	Sensitivity (%)	Specificity (%)
Xp11.2/TFE3 RCC vs ccRCC			
Calcification	0.717 (0.563–0.871)	50.0 (26.0–74.0)	93.3 (83.8–98.2)
Peak enhancement	0.836 (0.731–0.941)	88.9 (65.3–98.6)	78.3 (65.8–87.9)
Calcification+ Peak enhancement	0.896 (0.812–0.980)	94.4 (72.7–99.9)	73.3 (60.3–83.9)
Xp11.2/TFE3 RCC vs pRCC			
Age	0.693 (0.536–0.849)	66.7 (41.0–86.7)	71.9 (53.3–86.3)
Peak enhancement	0.708 (0.554–0.863)	66.7 (41.0–86.7)	75.0 (56.6–88.5)
Age + Peak enhancement	0.786 (0.641–0.932)	50.0 (26.0–74.0)	100.0 (89.1–100.0)

## Discussion

Xp11.2/TFE3 RCC is a rare form of RCC, which is characterized by several and various translocations involving chromosome Xp11.2, resulting in TFE3 gene fusion [15]. It was found that Xp11.2/TFE3 RCC tended to affect younger people. This study is the first to investigate the CEUS findings of Xp11.2/TFE3 RCC compared with those of ccRCC and pRCC in adult patients using a relatively large sample.

In our study, the age of the patients with Xp11.2/TFE3 RCC was relatively younger, ranging from 20 to 68 years with a mean age of 38.3 ± 16.3 years, and 61.1% were younger than 40 years old. Xp11.2/TFE3 RCC more frequently affected women. The ratio of male-to-female was 8:10, the female has slight predominance, this finding is consistent with previous reports [16, 17]. Compared with ccRCC and pRCC, there was a significant difference in age and gender in the univariate analysis. Thus, the presence of RCC in a young female adult should increase the suspicion of Xp11.2/TFE3 RCC. The most common symptoms of Xp11.2/TFE3 RCC include abdominal pain, flank mass, and gross haematuria, which are nonspecific [18]. In our study, these symptoms were present in 38.9% (7/18) of the patients, and the other 61.1% (11/18) of patients were detected incidentally. Compared with paediatric patients, adult patients with Xp11.2/TFE3 RCC usually have a more invasive clinical course and a poorer prognosis [19]. In our study, 3 patients with Xp11.2/TFE3 RCC were found to have recurrence or metastases in short term follow-up with a mean interval of 17.9 months.

Some conventional US and CEUS findings may help clinicians to consider the possibility of Xp11.2/TFE3 RCC; on conventional US, Xp11.2/TFE3 RCC may present as a cystic solid mixed mass [7]. In our study, the cystic component was present in 5 cases (27.8%). Furthermore, most cases (66.7%) showed heterogeneous enhancement on CEUS, which was similar to the findings of previous studies [20, 21]. This feature is related to pathological changes in the tumour, such as haemorrhage, necrosis, or cystic changes inside the tumour, and may reflect heterogeneous components [22, 23]. Calcification was recognized as an important sign of Xp11.2/TFE3 RCC in many previous reports [23, 24]. In our study, calcifications were detected in 50% of Xp11.2/TFE3 RCC cases and were more common in Xp11.2/TFE3 RCC than in ccRCC or pRCC; the calcifications appeared in a circular arrangement around or within the tumour (Fig. 1), which was in agreement with the findings of a previous report [21]. On CEUS imaging, the fast or iso wash-in mode generally indicates that there are multiple large-calibre feeding vessels in the tumour, and the fast or iso wash-out mode indicates that the tumour has arteriovenous shunt [25]. In our study, 61.1% of cases showed simultaneous wash-in, and 88.9% of cases showed fast- or simultaneous-out. As a hypovascular tumour, Xp11.2/TFE3 RCC was reported to display a lower enhancement degree than the normal renal cortex in contrast-enhanced studies [26, 27]. In our study, at peak enhancement, 6 cases (33.3%) showed hypoenhancement, 10 cases (55.6%) showed isoenhancement, and 2 cases (11.1%) showed hyperenhancement. Previous studies reported that the presence of a pseudocapsule was a pathologic feature often observed in the early stage of RCCs or in low-grade RCCs [28], the incidence of pseudocapsule in Xp11.2/TFE3 RCC reached 63.6% [29], while in our study, a pseudocapsule was present in only 6 cases (33.3%).

ccRCCs are hypervascular tumours and more frequently affect older men. The conventional US and CEUS features include intratumoural cysts, hyperenhancement in early phase, wash-out in the late phase, presence of pseudocapsule, and heterogeneous enhancement, which increase with tumour size [30]. However, some of the imaging features can overlap with those of Xp11.2/TFE3 RCC. In our multivariate logistic regression analysis, we found two significant predictors for differentiating Xp11.2/TFE3 RCC from ccRCC. The presence of calcification and lower peak enhancement were more likely in Xp11.2/TFE3 RCC. Calcification in ccRCC is less frequent than that in other subtypes of RCC [31], and the enhancement in ccRCCs is significantly greater than that in the other subtypes [32]. Therefore, the presence of calcification and lower peak enhancement are valuable clues for differentiating between Xp11.2/TFE3 RCC and ccRCC. In our study, the calcification combined peak enhancement model had good diagnostic performance in the differential diagnosis of Xp11.2/TFE3 RCC from ccRCC.

PRCCs typically appear hypovascular and are commonly seen in patients over 55 years of age [1]. On CEUS imaging, slow wash-in, fast wash-out and homogeneous hypoenhancement at the peak are considered to be the more common characteristic of pRCC, which could be helpful to differential diagnose pRCC from Xp11.2/TFE3 RCC [33, 34]. Some poorly differentiated pRCCs, such as those with type 2 pathology and a large size, may present with heterogeneous enhancement due to necrosis or haemorrhage within the tumour [35], therefore it is difficult to be differential diagnosis from Xp11.2/TFE3 RCC. In our study, calcification was less frequent in pRCC than in Xp11.2/TFE3 RCC; however, pRCC also showed calcification more often than ccRCC [31]. Thus, the value of this characteristic for differentiating between Xp11.2/TFE3 RCC and pRCC remains questionable. Although both tumours seem to be hypovascular, a previous study showed that the attenuation values of Xp11.2/TFE3 RCC in the corticomedullary phase and early excretory phase were higher than that of pRCC [20]. In our study, most cases (55.6%) showed isoenhancement, the peak enhancement of Xp11.2/TFE3 RCC was relatively higher than that of pRCC. In our multivariate logistic regression analysis, we found two significant predictors for differentiating Xp11.2/TFE3 RCC from pRCC: younger age and relatively higher peak enhancement were more likely to be present in Xp11.2/TFE3 RCC. The age combined peak enhancement model had good diagnostic performance in the differential diagnosis of Xp11.2/TFE3 RCC from pRCC.

This study had some limitations. First, Xp11.2/TFE3 RCC is a very rare, and this investigation was limited because of its small sample size and the nature of a retrospective study. Second, only two subtypes of non-Xp11.2/TFE3 RCC were used in this study, and some other subtypes of renal tumours are needed for comparison in further studies. Third, the quantitative measurement of peak enhancement may provide further valuable information regarding the differentiation between Xp11.2/TFE3 RCC and the other subtypes of RCC in the future.

## Conclusions

Our study suggests that young age with the presence of calcification, simultaneous wash-in, fast or simultaneous wash-out, iso- or hypoenhancement peak enhancement and heterogeneous enhancement on CEUS are helpful for the diagnosis of Xp11.2/TFE3 RCC. The presence of calcification and lower peak enhancement are more likely to be present in Xp11.2/TFE3 RCC than in ccRCC; moreover, compared with pRCC, younger age and relatively high peak enhancement are more likely to be present in Xp11.2/TFE3 RCC.

## Supplementary information

**Additional file 1.** Characteristics of patients with Xp11.2/TFE3 RCC. The clinical characteristics of the 18 patients with Xp11.2/TFE3 RCC.

**Additional file 2.** The interreader agreement of conventional US and CEUS features in the three subtypes of RCC for Reader 1 and Reader 2. The weighted kappa value that interprets the concordance between the interobserver agreement for these US and CEUS features ranged from 0.61 to 0.89.

## Data Availability

The datasets used and/or analysed during the current study are available from the corresponding author upon reasonable request.

## Abbreviations

AUC: Area under the curve
ccRCC: Clear cell renal cell carcinoma
CDFI: Colour Doppler flow imaging
CEUS: Contrast-enhanced ultrasound
CIs: Confidence intervals
CPS: Contrast pulse sequence
CT: Computed tomography
FISH: Fluorescence in situ hybridization
MRI: Magnetic resonance imaging
ORs: Odds ratios
pRCC: Papillary renal cell carcinoma
RCC: Renal cell carcinoma
ROC: Receiver operating characteristic
UWN: Ultra-wideband nonlinearity
WHO: World Health Organization
Xp11.2/TFE3 RCC: Renal cell carcinoma associated with Xp11.2 translocation/TFE3 gene fusion

## References

[1] Lopez-Beltran A, Scarpelli M, Montironi R, Kirkali Z (2006). 2004 WHO classification of the renal tumors of the adults. Eur Urol.

[2] Kmetec A, Jeruc J (2014). Xp 11.2 translocation renal carcinoma in young adults; recently classified distinct subtype. Radiol Oncol.

[3] Kuroda N, Mikami S, Pan CC, Cohen RJ, Hes O, Michal M (2012). Review of renal carcinoma associated with Xp11.2 translocations/TFE3 gene fusions with focus on pathobiological aspect. Histol Histopathol.

[4] He J, Huan Y, Qiao Q, Zhang J, Zhang JS (2014). Renal carcinomas associated with Xp11.2 translocations: are CT findings suggestive of the diagnosis?. Clin Radiol.

[5] Dai C, Sheng R, Ding Y, Yang M, Hou J, Zhou J (2019). Magnetic resonance imaging findings of renal cell carcinoma associated with Xp11.2 translocation/TFE3 gene fusion in adults: a pilot study. Abdom Radiol.

[6] Gerst S, Hann LE, Li D, Gonen M, Tickoo S, Sohn MJ (2011). Evaluation of renal masses with contrast-enhanced ultrasound: initial experience. AJR Am J Roentgenol.

[7] Ling W, Ma X, Luo Y, Chen L, Wang H, Wang X (2017). Ultrasonographic findings of renal cell carcinomas associated with Xp11.2 translocation/TFE3 gene fusion. Contrast Media Mol Imaging.

[8] Wei SP, Xu CL, Zhang Q, Zhang QR, Zhao YE, Huang PF (2017). Contrast-enhanced ultrasound for differentiating benign from malignant solid small renal masses: comparison with contrast-enhanced CT. Abdom Radiol (NY).

[9] Harvey CJ, Alsafi A, Kuzmich S, Ngo A, Papadopoulou I, Lakhani A (2015). Role of US contrast agents in the assessment of indeterminate solid and cystic lesions in native and transplant kidneys. Radiographics..

[10] Piscaglia F, Nolsøe C, Dietrich CF, Cosgrove DO, Gilja OH, Bachmann Nielsen M (2012). The EFSUMB guidelines and recommendations on the clinical practice of contrast enhanced ultrasound (CEUS): update 2011 on nonhepatic applications. Ultraschall Med.

[11] Bertolotto M, Bucci S, Valentino M, Currò F, Sachs C, Cova MA (2018). Contrast-enhanced ultrasound for characterizing renal masses. Eur J Radiol.

[12] Xue LY, Lu Q, Huang BJ, Li Z, Li CX, Wen JX (2015). Papillary renal cell carcinoma and clear cell renal cell carcinoma: differentiation of distinct histological types with contrast-enhanced ultrasonography. Eur J Radiol.

[13] Jiang J, Chen Y, Zhou Y, Zhang H (2010). Clear cell renal cell carcinoma: contrast-enhanced ultrasound features relation to tumor size. Eur J Radiol.

[14] Ascenti G, Gaeta M, Magno C, Mazziotti S, Blandino A, Melloni D (2004). Contrast-enhanced second-harmonic sonography in the detection of pseudocapsule in renal cell carcinoma. AJR Am J Roentgenol.

[15] Rao Q, Williamson SR, Zhang S, Eble JN, Grignon DJ, Wang M (2013). TFE3 break-apart FISH has a higher sensitivity for Xp11.2 translocation-associated renal cell carcinoma compared with TFE3 or cathepsin K immunohistochemical staining alone: expanding the morphologic spectrum. Am J Surg Pathol.

[16] Rao Q, Chen JY, Wang JD, Ma HH, Zhou HB, Lu ZF (2011). Renal cell carcinoma in children and young adults: clinicopathological, immunohistochemical, and VHL gene analysis of 46 cases with follow-up. Int J Surg Pathol.

[17] Wang W, Ding J, Li Y, Wang C, Zhou L, Zhu H (2014). Magnetic resonance imaging and computed tomography characteristics of renal cell carcinoma associated with Xp11.2 translocation/TFE3 gene fusion. PLoS One.

[18] Hung CC, Pan CC, Lin CC, Lin AT, Chen KK, Chang YH (2011). XP11.2 translocation renal cell carcinoma: clinical experience of Taipei veterans general hospital. J Chin Med Assoc.

[19] Klatte T, Streubel B, Wrba F, Remzi M, Krammer B, de Martino M (2012). Renal cell carcinoma associated with transcription factor E3 expression and Xp11.2 translocation: incidence, characteristics, and prognosis. Am J Clin Pathol.

[20] Woo S, Kim SY, Lee MS, Moon KC, Kim SH, Cho JY (2015). MDCT findings of renal cell carcinoma associated with Xp11.2 translocation and TFE3 gene fusion and papillary renal cell carcinoma. AJR Am J Roentgenol.

[21] He J, Gan W, Liu S, Zhou K, Zhang G, Guo H (2015). Dynamic computed tomographic features of adult renal cell carcinoma associated with Xp11.2 translocation/TFE3 gene fusions: comparison with clear cell renal cell carcinoma. J Comput Assist Tomogr.

[22] Armah HB, Parwani AV (2010). Xp11.2 translocation renal cell carcinoma. Arch Pathol Lab Med.

[23] Koo HJ, Choi HJ, Kim MH, Cho KS (2013). Radiologic-pathologic correlation of renal cell carcinoma associated with Xp11.2 translocation. Acta Radiol.

[24] Liu K, Xie P, Peng W, Zhou Z (2014). Renal carcinomas associated with Xp11.2 translocations/TFE3 gene fusions: findings on MRI and computed tomography imaging. J Magn Reson Imaging.

[25] Wei S, Fu N, Yao C, Liu P, Yang B (2013). Two- and three-dimensional contrast-enhanced sonography for assessment of renal tumor vasculature: preliminary observations. J Ultrasound Med.

[26] Kato H, Kanematsu M, Yokoi S, Miwa K, Horie K, Deguchi T (2011). Renal cell carcinoma associated with Xp11.2 translocation/ TFE3 gene fusion: radiological findings mimicking papillary subtype. J Magn Reson Imaging.

[27] Zhu QQ, Wang ZQ, Zhu WR, Chen WX, Wu JT (2013). The multislice CT findings of renal carcinoma associated with XP11.2 translocation/TFE gene fusion and collecting duct carcinoma. Acta Radiol.

[28] Tsili AC, Argyropoulou MI, Gousia A, Kalef-Ezra J, Sofikitis N, Malamou-Mitsi V (2012). Renal cell carcinoma: value of multiphase mdct with multiplanar reformations in the detection of pseudocapsule. AJR Am J Roentgenol.

[29] Cheng X, He J, Gan W, Fan X, Yang J, Zhu B (2015). Pseudocapsule of renal cell carcinoma associated with Xp11.2 translocation/TFE3 gene fusion: a clue for tumor enucleation?. Int J Clin Exp Pathol.

[30] Gulati M, King KG, Gill IS, Pham V, Grant E, Duddalwar VA (2015). Contrast-enhanced ultrasound (CEUS) of cystic and solid renal lesions: a review. Abdom Imaging.

[31] Kim JK, Kim TK, Ahn HJ, Kim CS, Kim KR, Cho KS (2002). Differentiation of subtypes of renal cell carcinoma on helical CT scans. AJR Am J Roentgenol.

[32] Young JR, Margolis D, Sauk S, Pantuck AJ, Sayre J, Raman SS (2013). Clear cell renal cell carcinoma: discrimination from other renal cell carcinoma subtypes and oncocytoma at multiphasic multidetector CT. Radiology..

[33] Sun D, Wei C, Li Y, Lu Q, Zhang W, Hu B (2016). Contrast-enhanced ultrasonography with quantitative analysis allows differentiation of renal tumor histotypes. Sci Rep.

[34] Li X, Liang P, Guo M, Yu J, Yu X, Cheng Z (2013). Real-time contrast-enhanced ultrasound in diagnosis of solid renal lesions. Discov Med.

[35] Yamada T, Endo M, Tsuboi M, Matsuhashi T, Takase K, Higano S (2008). Differentiation of pathologic subtypes of papillary renal cell carcinoma on CT. AJR Am J Roentgenol.

