# Supplementary material for: Contrast-enhanced ultrasound findings of adult renal cell carcinoma associated with Xp11.2 translocation/TFE3 gene fusion: comparison with clear cell renal cell carcinoma and papillary renal cell carcinoma
Source: Cancer Imaging. 2019 Dec 31;20:1. doi: 10.1186/s40644-019-0268-7 (PMC6938633; doi:10.1186/s40644-019-0268-7)
Supplement: Supplementary file 1 — Additional file 1. Characteristics of patients with Xp11.2/TFE3 RCC. The clinical characteristics of the 18 patients with Xp11.2/TFE3 RCC. [file 40644_2019_268_MOESM1_ESM.docx]

Additional file 1. Characteristics of patients with Xp11.2/TFE3 RCC

| Case | Age (y)/ gender | Tumor side | Tumor size (cm) | Clinical presentation | Operation | Follow-up (months) | Recurrence or metastases |
| --- | --- | --- | --- | --- | --- | --- | --- |
| 1 | 33/M | R | 4.5 | Incidentally detected on US | LNSN | 46 | No |
| 2 | 21/F | R | 4.0 | Gross hematuria, Flank pain | LRN | 16 | Retroperitoneal space, Nephrectomy bed |
| 3 | 20/F | L | 4.3 | Flank pain, fever | LRN | 5 | No |
| 4 | 20/F | R | 6.1 | Gross hematuria | ORN+ VCTER | 18 | Liver (detected 6 months postoperation), lung and retroperitoneal space (detected 13 months postoperation) |
| 5 | 59/F | L | 2.3 | Flank pain | LRN | 32 | No |
| 6 | 20/F | R | 6.3 | Incidentally detected on US | RLNSN | 7 | Nephrectomy bed |
| 7 | 52/F | R | 4.5 | Incidentally detected on US | RLNSN | Lost | - |
| 8 | 26/F | L | 6.6 | Incidentally detected on US | RLNSN | Lost | - |
| 9 | 30/M | R | 5.6 | Incidentally detected on US | LRN | 52 | No |
| 10 | 68/M | R | 2.5 | Flank pain | LRN | 10 | No |
| 11 | 35/F | R | 1.7 | Incidentally detected on CT | LNSN | 14 | No |
| 12 | 24/F | R | 4.2 | Incidentally detected on US | LRN | 7 | No |
| 13 | 36/M | L | 5.1 | Flank pain | LRN | 9 | No |
| 14 | 60/M | L | 2.7 | Incidentally detected on CT | LNSN | 6 | No |
| 15 | 47/F | L | 5.6 | Incidentally detected on US | LRN | Lost | - |
| 16 | 27/M | R | 3.8 | Incidentally detected on US | LNSN | 36 | No |
| 17 | 57/M | R | 5.1 | Gross hematuria, fever | LRN | 5 | No |
| 18 | 54/M | L | 2.4 | Incidentally detected on CT | LNSN | 6 | No |

Abbreviations: M=male; F=female; R=right; L=left, ORN, open radical nephrectomy; RLNSN, robotic-assisted laparoscopic nephron-sparing nephrectomy; LRN, laparoscopic radical ne­phrectomy; LNSN, laparoscopic nephron-sparing nephrectomy; VCTER: vena cava tumor embolus resection
