# Supplementary material for: Contrast-enhanced ultrasound findings of adult renal cell carcinoma associated with Xp11.2 translocation/TFE3 gene fusion: comparison with clear cell renal cell carcinoma and papillary renal cell carcinoma
Source: Cancer Imaging. 2019 Dec 31;20:1. doi: 10.1186/s40644-019-0268-7 (PMC6938633; doi:10.1186/s40644-019-0268-7)
Supplement: Supplementary file 2 — Additional file 2. The interreader agreement of conventional US and CEUS features in the three subtypes of RCC for Reader 1 and Reader 2. The weighted kappa value that interprets the concordance between the interobserver agreement for these US and CEUS features ranged from 0.61 to 0.89. [file 40644_2019_268_MOESM2_ESM.docx]

Additional file 2. The interreader agreement of conventional US and CEUS features in the three subtypes of RCC for Reader 1 and Reader 2

| Features | Xp11.2/TFE3 RCC  (n=18) | |  | ccRCC  (n=60) | |  | pRCC  (n=32) | |
| --- | --- | --- | --- | --- | --- | --- | --- | --- |
|  | Consensus (R1, R2) | Kappa  (95% CI) |  | Consensus (R1, R2) | Kappa  (95% CI) |  | Consensus (R1, R2) | Kappa  (95% CI) |
| Echogenicity |  | 0.77(0.53-1.0) |  |  | 0.83(0.72-0.94) |  |  | 0.84(0.66-1.0) |
| Hypoechoic | 6(6,4) |  |  | 28(28,28) |  |  | 17(19,19) |  |
| Isoechoic | 8(8,11) |  |  | 21(22,22) |  |  | 13(12,11) |  |
| Hyperechoic | 4(4,3) |  |  | 11(10,10) |  |  | 2(1,2) |  |
| Cystic component |  | 0.85(0.57-1.0) |  |  | 0.85(0.72-0.99) |  |  | 0.71(0.42-1.0) |
| Present | 5(4,5) |  |  | 23(19,23) |  |  | 8(5,8) |  |
| Absent | 13(14,13) |  |  | 37(41,37) |  |  | 24(27,24) |  |
| Calcification |  | 0.89(0.68-1.0) |  |  | 0.73(0.38-1.0) |  |  | 0.61(0.22-1.0) |
| Present | 9(10,9) |  |  | 4(3,5) |  |  | 5(5,4) |  |
| Absent | 9(8,9) |  |  | 56(57,55) |  |  | 27(27,28) |  |
| Color flow signal |  | 0.78(0.50-1.0) |  |  | 0.68(0.49-0.87) |  |  | 0.80(0.53-1.0) |
| Present | 7(7,9) |  |  | 37(38,37) |  |  | 7(5,7) |  |
| Absent | 11(11,9) |  |  | 23(22,23) |  |  | 25(27,25) |  |
| Wash-in(%) |  | 0.78(0.48-1.0) |  |  | 0.79(0.40-1.0) |  |  | 0.83(0.61-1.0) |
| Slow-in | 7(8,8) |  |  | 3(3,2) |  |  | 23(23,25) |  |
| Simultaneous-in | 11(10,10) |  |  | 57(57,58) |  |  | 9(9,7) |  |
| Peak Enhancement |  | 0.82(0.60-1.0) |  |  | 0.78(0.62-0.94) |  |  | 0.89(0.73-1.0) |
| Hypoenhancement | 6(5,7) |  |  | 3(3,3) |  |  | 24(24,26) |  |
| Isoenhancement | 10(11,9) |  |  | 10(12,10) |  |  | 4(4,2) |  |
| Hyperenhancement | 2(2,2) |  |  | 47(45,47) |  |  | 4(4,4) |  |
| Homogeneity |  | 0.75(0.44-1.0) |  |  | 0.87(0.74-1.0) |  |  | 0.75(0.52-0.98) |
| Homogeneous | 6(5,7) |  |  | 17(16,16) |  |  | 19 (17,19) |  |
| Heterogeneous | 12(13,11) |  |  | 43(44,44) |  |  | 13(15,13) |  |
| Wash-out |  | 0.64(0.00-1.0) |  |  | 0.83(0.67-0.99) |  |  | 0.65(0.02-1.0) |
| Fast- or simultaneous-out | 16(16,17) |  |  | 45(44,44) |  |  | 31(31,30) |  |
| Slow-out | 2(2,1) |  |  | 15(16,16) |  |  | 1(1,2) |  |
| Pseudocapsule |  | 0.75(0.44-1.0) |  |  | 0.87(0.74-0.99) |  |  | 0.81(0.61-1.0) |
| Present | 6(5,7) |  |  | 26(26,28) |  |  | 16(17,16) |  |
| Absent | 12(13,11) |  |  | 34(34,32) |  |  | 16(15,16) |  |
